# Serum Bilirubin Levels and Promoter Variations in *HMOX1* and *UGT1A1* Genes in Patients with Fabry Disease

**DOI:** 10.1155/2017/9478946

**Published:** 2017-08-16

**Authors:** Alena Jirásková, Giulia Bortolussi, Gabriela Dostálová, Lenka Eremiášová, Lubor Golaň, Vilém Danzig, Aleš Linhart, Libor Vítek

**Affiliations:** ^1^Department of Medical Biochemistry and Laboratory Diagnostics, 1st Faculty of Medicine, Charles University, Katerinska 32, 12000 Prague 2, Czech Republic; ^2^2nd Department of Internal Medicine and Department of Cardiovascular Medicine, 1st Faculty of Medicine, Charles University, Katerinska 32, 12000 Prague 2, Czech Republic; ^3^4th Department of Internal Medicine, 1st Faculty of Medicine, Charles University, Katerinska 32, 12000 Prague 2, Czech Republic

## Abstract

The aim of our study was to assess the possible relationships among heme oxygenase (HMOX), bilirubin UDP-glucuronosyl transferase (UGT1A1) promoter gene variations, serum bilirubin levels, and Fabry disease (FD). The study included 56 patients with FD (M : F ratio = 0.65) and 185 healthy individuals. Complete standard laboratory and clinical work-up was performed on all subjects, together with the determination of total peroxyl radical-scavenging capacity. The (GT)n and (TA)n dinucleotide variations in the *HMOX1* and *UGT1A1* gene promoters, respectively, were determined by DNA fragment analysis. Compared to controls, patients with FD had substantially lower serum bilirubin levels (12.0 versus 8.85 *μ*mol/L, *p* = 0.003) and also total antioxidant capacity (*p* < 0.05), which showed a close positive relationship with serum bilirubin levels (*p* = 0.067) and the use of enzyme replacement therapy (*p* = 0.036). There was no association between *HMOX1* gene promoter polymorphism and manifestation of FD. However, the presence of the TA_7_ allele *UGT1A1* gene promoter, responsible for higher systemic bilirubin levels, was associated with a twofold lower risk of manifestation of FD (OR = 0.51, 95% CI = 0.27–0.97, *p* = 0.038). Markedly lower serum bilirubin levels in FD patients seem to be due to bilirubin consumption during increased oxidative stress, although *UGT1A1* promoter gene polymorphism may modify the manifestation of FD as well.

## 1. Introduction

Fabry disease (FD, OMIM 301500), an X-linked metabolic disorder, is caused by a deficiency of the lysosomal enzyme *α*-galactosidase A, resulting in the accumulation of glycosphingolipids (in particular globotriaosylceramide (GB3)) in endothelial cells and other tissues [1, 2]. The majority of FD complications results from progressive damage to the central and peripheral nervous system, kidney, and heart due to severe vasculopathy [2, 3]. Indeed, *α*-galactosidase A deficiency was shown to accelerate atherosclerosis in apolipoprotein E-deficient mice [4]. Moreover, endothelial dysfunction was also reported in a clinical study on FD patients [5], but the vascular damage in FD differs from classical atherosclerosis [6–8]. The storage in FD, predominantly found in endothelial and smooth muscle cells as well as in stenotic lesions, is characterized by intimal and subintimal proliferation [6]. Increasing clinical evidence suggest that FD is a rather heterogeneous disease differing in the age of manifestation, severity, and organ involvement depending on specific genetic background [9]. There is also an increasing evidence that FD prevalence may be largely underestimated [9, 10], which strongly supports the importance of known and reasons for additional putative genetic factors influencing its clinical manifestation.

The pathological processes in FD are associated with increased oxidative, nitrosative, and carbonyl stress as evidenced by excessive production of reactive oxygen species (ROS) [11–13]; increased plasma and urinary carbonylated proteins [14]; and nitrotyrosine in plasma, cardiac, and vascular tissues [15–17], resulting in DNA oxidative damage in the cells and tissues [12, 17].

The oxidative stress defense system consists of antioxidative enzymes and antioxidative substrates. Among the latter, bilirubin, the heme catabolic product, belongs among the most potent endogenous antioxidants [18, 19]. In fact, serum bilirubin positively correlates with total antioxidant status (TAS) in newborns with neonatal jaundice, as well as adult subjects (for review, see [20]). Bilirubin has been reported to be a strong negative predictor/biomarker of oxidative stress-mediated diseases such as atherosclerosis [21]. On the other hand, decreased systemic antioxidant defense mechanisms have been reported in patients with FD [13, 14].

By regulating bilirubin production and its biotransformation in the liver, heme oxygenase-1 (encoded by *HMOX1*, OMIM^∗^141250) and bilirubin UDP-glucuronosyl transferase (*UGT1A1*, OMIM^∗^191740) enzymes play an important role in oxidative stress defense. Microsatellite variations in the promoter regions modulate *HMOX1* and *UGT1A1* gene expression and have been associated with oxidative stress-mediated diseases such as atherosclerosis [22, 23]. Indeed, this is true for subjects with a high number of (GT)n repetitions in the *HMOX1* gene promoter, resulting in decreased expression of HMOX1 (categorized as class L allele carriers) [22]. On the other hand, those with congenitally decreased expression of *UGT1A1* (which is caused in the majority of Caucasians by homozygosity of the so-called UGT1A1^∗^28 allele) [24] results in mild unconjugated hyperbilirubinemia (Gilbert's syndrome), a condition associated with a decreased risk of cardiovascular diseases [21].

The objective of this study was to determine whether genetic variations in *HMOX1* and *UGT1A1* genes, as well as systemic levels of bilirubin, might affect the risk of the development of FD, a metabolic disorder accompanied by increased oxidative stress.

## 2. Materials and Methods

### 2.1. Patients

The study was performed on 56 FD patients (M : F ratio = 0.70) diagnosed in our center from 2000 to 2012. The diagnosis of FD was based on a demonstrated reduction of *α*-galactosidase A activity in leukocytes or plasma and confirmed by DNA mutation analysis. Of those, 45% suffered from cardiovascular disease (*n* = 25), 48% from neurological complications (*n* = 27), 30% from renal disease (*n* = 17), 27% from cutaneous disease (*n* = 15), and 18% from ocular complications (*n* = 10). Out of the whole FD group, 55% of patients (*n* = 31) were treated with ERT according to international recommendations [25].

The control group was based upon age- and sex-matched 185 clinically healthy subjects without any chronic medication, representing a general population sample from the same geographical region. These subjects were recruited from healthy blood donors and employees of the General University Hospital. All subjects in both cohorts were of Caucasian ancestry.

The study protocol and all procedures conformed to the ethical guidelines of the 1975 Declaration of Helsinki, as reflected in a priori approval by the institution's Ethics Committee, and all subjects signed informed-consent forms.

### 2.2. Laboratory Investigation

Peripheral venous blood was obtained from all participants after overnight fasting. The serum was immediately processed, and bilirubin was determined on an automatic analyzer (modular analyzer, Roche Diagnostics GmbH, Germany), using routine laboratory assay.

Total antioxidant status (TAS) was determined fluorometrically as the serum peroxyl radical-scavenging capacity, based on the relative proportion of chain-breaking antioxidant consumption present in the serum compared to that of Trolox (a reference and calibration antioxidant compound) [26].

The (GT)_n_ (dbSNP rs1805173) and (TA)_n_ (dbSNP rs81753472) dinucleotide variations in *HMOX1* and *UGT1A1* gene promoters, respectively, were determined by fragment analysis using an automated capillary DNA sequencer, as previously described [27]. The length variations of *HMOX1* (GT)_n_ repeats were classified into short S (*n* < 27), medium M (*n* = 27–32), and long L (*n* ≥ 33) subgroups.

### 2.3. Statistical Analyses

The data are presented as either the mean ± SD or the median and 25–75% interquartile range. Data were evaluated by a *t*-test or the Rank Sum test depending on their normality. Allele frequency was analyzed by a chi-square test. Linear regression analyses were used to assess the association between serum bilirubin and TAS. The impact of individual genetic variations on the risk of FD was analyzed by logistic regression. All tests were made at the 0.05 significance level. All statistical analyses were performed using SigmaPlot software, version 11.0 (Systat Software Inc., USA).

## 3. Results

### 3.1. Serum Bilirubin Concentrations and Total Antioxidant Status in Patients with FD

**Figure 1:**
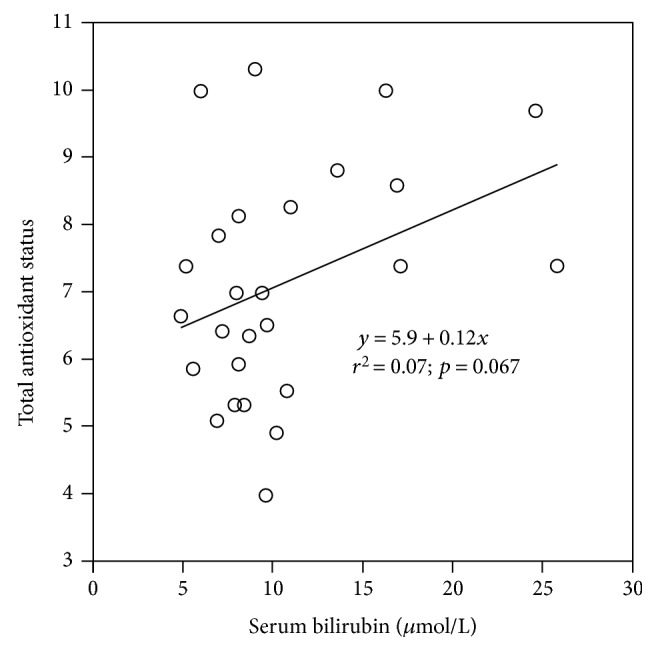
The relationship between serum bilirubin and total antioxidant status in patients with FD. Each dot represents a single subject. FD, Fabry disease.

Compared to age-matched control subjects, patients with FD displayed significantly lower systemic bilirubin concentrations (Table 1). The difference was more evident when subjects were gender split; in fact, only FD-affected females showed significantly lower systemic bilirubin concentrations compared to healthy controls (Table 1). On the contrary, the difference was not evident in male subjects, probably due to the positive effect of ERT (predominantly used in male FD patients), which was associated with significantly higher serum bilirubin levels (Table 2). Similarly, the total serum peroxyl-scavenging activity, depressed in FD patients, was also significantly improved in ERT-treated patients (Table 2). Both variables (i.e., TAS and serum bilirubin levels) were positively correlated with borderline statistical significance (*p* = 0.067, Figure 1). No differences in serum bilirubin levels were observed when analyzing FD patient subgroups according to organ involvement (data not shown).

### 3.2. The Association between *HMOX1* and *UGT1A1* Promoter Gene Variants and FD Manifestation

As expected, significantly lower serum bilirubin concentrations between FD patients and control subjects were observed within all *UGT1A1* genotypes, with the lowest values in *UGT1A1* [TA]_6/6_ wild types and the highest in *UGT1A1* [TA]_7/7_ Gilbert's syndrome genotypes (Table 3). The *HMOX1* promoter gene status had no effect on serum bilirubin concentrations (data not shown).

No significant differences in frequencies of the L-allele in the *HMOX1* gene (associated with lower enzyme activity) between the FD and control groups were found (OR = 1.69; 95% CI = 0.54–5.24, *p* = 0.46) indicating no modifying role of *HMOX1* promoter gene variation in FD manifestation (Table 4). In contrast, frequency of the TA_7_ allele of the *UGT1A1* gene, responsible for higher serum bilirubin levels, was associated with a decreased risk of FD manifestation (OR = 0.51, 95% CI = 0.27–0.97, *p* = 0.038) (Table 4).

## 4. Discussion

Bilirubin has been reported to be a strong negative predictor/biomarker of oxidative stress-mediated diseases. This association has been reported for atherosclerosis and cancer, as well as metabolic, autoimmune, and neurodegenerative diseases [20, 24, 28].

With respect to oxidative stress-mediated damage, FD is not the exception, and the role of increased oxidative, nitrosative, and carbonyl stress in pathogenesis of cardiovascular complications of FD is indisputable [11, 14, 15, 17].

Indeed, increased protein nitration and oxidative DNA damage leading to accelerated apoptosis were reported in cardiomyocytes of FD patients [12, 17]. Moreover, increased lipoperoxidation and oxidative protein damage in the plasma of FD patients were reported by Biancini et al. [14]. The severity of FD correlated well with exaggeration of oxidative stress, as demonstrated in cardiomyocytes from male and female FD patients [17]. These observations are in line with *in vitro* data showing that GB3 accumulation within cultured endothelial cells derived from FD patients leads dose dependently to increased production of ROS [11].

In fact, low serum TAS was also observed in our FD patient cohort, together with significantly lower serum bilirubin concentrations. Since bilirubin is one of the most potent endogenous antioxidants [18, 19, 21], it was not surprising that TAS in our FD patients correlated well with serum bilirubin concentrations.

Increased consumption of antioxidant substrates seems to account for this observation, and this explanation is also supported by the decreased systemic ascorbate levels in FD patients reported by Moore et al. [29]. However, genetic factors predisposing to impaired oxidative stress defense are likely to play a role as well, as demonstrated by the protective effect of the UGT1A1^∗^28 allele observed in our study.

Although this observation may seem surprising, even monogenic diseases can be substantially influenced by genes capable of modifying disease phenotype [30]. In fact, this phenomenon was reported also in FD patients for polymorphisms of the genes coding for interleukin 6, endothelial nitric oxide synthase, factor V, protein Z [31], or those within the mitochondrial genome [32]. Hence, it appears that low serum bilirubin levels observed in FD patients reflect not only increased oxidative stress but also to a certain extent a genetic predisposition.

The beneficial impact of ERT on the amelioration of increased oxidative/nitrosative stress is supported by the observation of a notable decrease of nitrotyrosine staining in the dermal blood vessels of FD patients on *α*-galactosidase A therapy [15]. Additionally, the salutary effect of ERT on urinary GB3 concentrations in FD patients was described by Biancini et al. [13], and improvement of the disturbed redox status of FD patients on ERT was also reported in another study by Moore et al. [29]. Importantly, ERT was demonstrated to attenuate endothelial dysfunction and improve markers of oxidative stress response in FD patients [5]. ERT was also reported to significantly improve impaired gene and protein expression profiles in a Fabry mouse model (in particular those related to oxidative stress defense), further accounting for the therapeutic effect of ERT [33]. These data are in line with our observation of marked improvement of TAS, as well as serum bilirubin of our FD patients treated with ERT.

Bilirubin not only is a potent antioxidant but also significantly affects nitric oxide production [34]. Thus, low systemic concentrations of bilirubin observed in our study fit into the increased nitrosative stress reported by other authors [15, 17].

Apart from the possible effects on redox status, bilirubin can also exert its protective effects through other mechanisms, such as improvement of endothelial dysfunction, known to be present in FD patients [11]. Indeed, bilirubin was reported to substantially ameliorate inflammation-induced dysfunction of endothelial adhesion molecules [35, 36]. In addition, FD is associated with proinflammatory status, as demonstrated in the plasma of FD patients by increased expression of the proinflammatory cytokines IL-1*β* and TNF-*α*, compared to normal controls [37] as well as levels of IL-6 and TNF-*α* [14], both cytokines being negatively correlated with bilirubin concentrations as shown in both experimental and clinical studies [38, 39].

We believe that marked disturbances in the oxidative stress defense system observed in our study might be of clinical importance and that they may have a prognostic value. Recently, we have shown that serum uric acid, an indirect indicator of oxidative stress, is associated with the long-term prognosis of FD patients [40]. In addition, therapeutic intervention based on intravenous administration of ascorbic acid was demonstrated to ameliorate vertebrobasilar hyperperfusion in patients with FD [29]. Thus, it is tempting to speculate whether modulation of the heme catabolic pathway to increase systemic levels of bilirubin [41] might improve the prognosis of FD patients.

## 5. Conclusions

FD is associated with markedly lower serum bilirubin levels, most likely due to bilirubin consumption during the increased oxidative stress associated with this disease. However, we propose that genetic factors also affecting the defense against increased oxidative stress (*UGT1A1* promoter gene polymorphism) may also modify the manifestation of FD.

## Figures and Tables

**Table 1:** Serum bilirubin concentrations in patients with FD.

	FD	Controls	*p* value
	Males + females		
Age [years]	**43 ± 11.5** (*n* = 56)	**41.7 ± 9.7** (*n* = 185)	0.303
Bilirubin [*μ*mol/L]	**9.0** [7.6–13.5]	**12.0** [9–16.4]	**0.006**
	Males		
Age [years]	**42 ± 11.6** (*n* = 22)	**41.6 ± 9.9** (*n* = 108)	0.77
Bilirubin [*μ*mol/L]	**13.3** [8.7–16.7]	**12.6** [9.8–17.7]	0.719
	Females		
Age [years]	**43.6 ± 17.1** (*n* = 34)	**41.9 ± 9.7** (*n* = 77)	0.313
Bilirubin [*μ*mol/L]	**8.1** [6.9–9.6]	**10.6** [8.5–14.9]	**0.002**

Data expressed as mean ± SD, or median and IQ range, depending on their normality. FD: Fabry disease.

**Table 2:** The effect of ERT on bilirubin and total antioxidant status in patients with FD.

	ERT+ (*n* = 31)	ERT− (*n* = 25)	Controls (*n* = 185)	*p* value
	Males + females			
Bilirubin [*μ*mol/L]	**9.7** [8.6–15.5]	**7.2** [5.6–8.7]		**0.004**
*TAS*	**7.14 ± 1.9**	**6.10 ± 1.2**		**0.036**
		**6.10 ± 1.2**	**6.92 ± 2**	**0.059**
	**7.14 ± 1.9**		**6.92 ± 2**	0.503
	Males^∗^			
*TAS*	**7.3 ± 2** (*n* = 21)	(*n* = 2)	**7.43 ± 1.9** (*n* = 108)	0.794
	Females			
Bilirubin [*μ*mol/L]	**8.9 ± 0.8** (*n* = 10)	**7.5 ± 1.9** (*n* = 23)		0.186
*TAS*	**6.5** [5.5–7.6]	**5.8** [5.2–7.4]		0.201
	**6.5** [5.5–7.6]		**6.0** [4.8–7.2] (*n* = 77)	0.235

Out of the entire group, 55% of patients were treated with ERT (enzyme replacement therapy) (95% men and 30% women). TAS, total antioxidant status, was determined according to [26]. FD: Fabry disease. ^∗^Only two male FD patients were untreated with ERT, making comparison with the untreated group impossible.

**Table 3:** Serum bilirubin concentrations in patients with FD and controls according to *UGT1A1* genotype.

*UGT1A1* genotype	FD	Controls	*p* value
6/6	**7.1** [6.1–10.7]	**10.2** [8.6–11.4]	**0.009**
6/7	**8.7** [7.1–11]	**12.4** [8.5–15.6]	**0.053**
7/7	**9.0** [6.4–15.2]	**17.8** [14.7–21.5]	**0.025**
*P for trend* ^∗^	0.538	**<0.001**	

Bilirubin expressed in *μ*mol/L as median and IQ range. FD: Fabry disease. ^∗^Differences in serum bilirubin concentrations across individual *UGT1A1* genotypes in FD and controls.

**Table 4:** The impact of variability of genes regulating heme catabolism on risk of FD.

	OR for risk of FD manifestation		*p* value
*HMOX1* (L status)	**1.69** [0.54–5.24]		0.46
*UGT1A1* [TA]_7_ status	**0.51** [0.27–0.97]		**0.038**

	FD	Controls	*p* value
*UGT1A1* [TA]_7_ allele frequency	**0.46**	**0.63**	**0.055**

FD: Fabry disease.

## Abbreviations

ERT: Enzyme replacement therapy
FD: Fabry disease
GB3: Globotriaosylceramide
HMOX: Heme oxygenase
ROS: Reactive oxygen species
TAS: Total antioxidant status
UGT1A1: Bilirubin UDP-glucuronosyl transferase.
